# Neutralizing Antibodies in Survivors of Sin Nombre and Andes Hantavirus Infection

**DOI:** 10.3201/eid1201.050930

**Published:** 2006-01

**Authors:** Francisca Valdivieso, Pablo Vial, Marcela Ferres, Chunyan Ye, Diane Goade, Analia Cuiza, Brian Hjelle

**Affiliations:** * Universidad del Desarrollo, Santiago, Chile;; † Pontifica Universidad Catolica de Chile, Santiago, Chile;; ‡ University of New Mexico Health Sciences Center, Albuquerque, New Mexico, USA;; § TriCore Reference Laboratory, Albuquerque, New Mexico USA

**Keywords:** hantavirus, Andes virus, neutralizing antibodies, Sin Nombre virus

## Abstract

We evaluated titers of homotypic and heterotypic neutralizing antibodies (NAbs) to Andes and Sin Nombre hantaviruses in plasma samples from 20 patients from Chile and the United States. All but 1 patient had high titers of NAb. None of the plasma samples showed high titers against the heterologous virus.

Hantavirus cardiopulmonary syndrome (HCPS), an emergent disease caused by New World hantaviruses, is associated with case-fatality ratios of 30% to 50%. Sin Nombre virus (SNV) and Andes virus are well-characterized hantavirus serotypes responsible for this disease in the southwestern United States and in the south cone of the Americas (Argentina, Brazil, and Chile), respectively (1). Since their recognition in 1993, they have caused hundreds of cases, many of them appearing in seasonal outbreaks. Other types of hantaviruses have been identified in the Americas during the past decade, causing diseases with variable severity. All of them are associated with different rodent hosts of the subfamily *Sigmodontinae*, family *Muridae*, and the distribution of each virus parallels that of the host (2).

No specific treatment for HCPS exists. Ribavirin, the only approved antiviral agent that is effective against hantaviruses in vitro (3), has shown efficacy in treating hemorrhagic fever with renal syndrome, a related disease that is caused by hantaviruses indigenous to the Old World (4). However, technical difficulties prevented a trial that was designed to evaluate the efficacy of ribavirin in treating HCPS from being completed (5).

Some evidence shows that neutralizing antibody (NAb) can affect the course of HCPS. Animal studies have shown that NAb confers passive protection from severe disease by Andes virus. Specifically, passive transfer of serum with high NAb titers from rhesus macaques vaccinated against Andes virus protected 100% of Syrian hamsters from lethal disease, even when administered 4–5 days after challenge with Andes virus (6). In humans, a high NAb titer on hospital admission is correlated with less severe HCPS (7)

Administering convalescent-phase plasma with a high NAb titer could be therapeutic in HCPS, as it is in other hemorrhagic fevers (8). In survivors of Sin Nombre infection, high titers of serum NAb could still be detected years after recovery, with no evidence of residual viral RNA in the plasma (9).

The severity of HCPS, the absence of effective treatment, its appearance in outbreaks and in case-clusters, and the potential use of hantaviruses as bioweapons have stimulated work toward hantavirus vaccine development. At present, an inactivated Hantaan virus vaccine is in use for persons at high risk for exposure to Old World hantaviruses, but its efficacy has recently been questioned (10). A DNA vaccine expressing the G1 and G2 glycoproteins encoded by the Hantaan virus M segment conferred sterilizing cross-protection against the other Old World hantaviruses, Seoul, Dobrava, and Puumala, in hamsters (11). For New World hantaviruses, in the hamster model for Andes disease, prior infection with widely disparate species conferred varying levels of cross-protection (12,13). Although these selected studies suggest some cross-protection among different hantavirus species, the considerable antigenic variation among members of the genus *Hantavirus* suggests that a monovalent vaccine will not likely confer sufficient protection for all of the pathogenic hantaviruses (14).

The persistence of NAb in plasma of survivors of Andes virus and SNV infections, as well as the in vitro cross-neutralization capacity of these NAbs against the heterotypic hantavirus, could have implications for use of convalescent-phase plasma to treat HCPS. For vaccine development, an evaluation of the duration of persistence of NAb and their cross-neutralization activities across different serotypes of hantaviruses would shed light upon the probability of obtaining satisfactory cross-protection among candidate vaccines against New World hantaviruses.

## The Study

We studied 20 serum samples from survivors of confirmed hantavirus infection, 11 from Chilean patients and 9 from patients in the southwestern United States. Samples were collected from 8 months to 11 years after the patient was hospitalized with HCPS. The neutralizing titer was measured for each sample against SNV and Andes virus by a focus-reduction neutralization assay in Vero E6 cells, as described previously (7). In brief, serial 2-fold dilutions of heat-inactivated patient plasma samples were made, from 1:100 to 1:1,600, and were mixed with equal volume of ≈50–100 focus-forming units per milliliter SNV (isolate SN77734, titer 2 × 10^6^/mL) or Andes virus (Chilean strain of human origin, isolate CHI-7913) and incubated at 37° for 1 hour (15). The mixture was then used to infect a confluent monolayer of Vero E6 cells (ATCC CRL 1586) in duplicate wells of a 48-well dish, with a 1.2% methylcellulose overlay in the medium to confine the virus to the foci. After incubation for 1 week, viral foci were detected with polyclonal rabbit anti-N antibody followed by peroxidase-conjugated goat anti-rabbit immunoglobulin G. Foci were enumerated under an inverted light microscope. NAb titers were defined as the reciprocal of the highest serum dilution that resulted in an 80% reduction in the number of foci compared to virus controls in duplicate assays.

The endpoint plasma NAb titers against Andes virus and SNV from Chilean and North American survivors of hantavirus infection are shown in the Table. All Chilean patients had detectable plasma NAb against Andes virus, with titers >1:400 in all but 1 patient. In contrast, 9 of the 11 samples failed to show NAb titers >1:100 against SNV, while the other 2 neutralized SNV only at low titers. Similarly, all North American patients had plasma NAb against SNV at titers >400, and only 1 showed some neutralization against Andes virus, at low titer. No relationship was seen between the endpoint NAb titers against the homotypic virus and time elapsed from acute disease in either Chilean or North American patients, nor did a particularly high homotypic titer predict that neutralizing activity would be present against the heterologous virus.

**Table:** Neutralizing antibody (NAb) titers against Andes virus (AND) and Sin Nombre virus (SNV) in survivors of hantavirus infection from Chile and the United States

Patient	Origin	Years after infection	AND NAb titer	SNV NAb titer
1	Chile	3	>1:1,600	1:100
2	Chile	4	1:400	<1:100
3	Chile	7	>1:1,600	<1:100
4	Chile	0.7	1:400	<1:100
5	Chile	4	1:400	<1:100
6	Chile	1	1:400	<1:100
7	Chile	4	1:800	1:100
8	Chile	3	1:200	<1:100
9	Chile	4	>1:1,600	<1:100
10	Chile	7	1:400	<1:100
11	Chile	1	1:400	<1:100
12	USA	1	<1:100	1:800
13	USA	3	<1:100	1:400
14	USA	4	<1:100	1:400
15	USA	4	1:100	>1:1,600
16	USA	3	<1:100	1:400
17	USA	5	<1:100	>1:1,600
18	USA	11	<1:100	1:400
19	USA	6	<1:100	1:800
20	USA	4	<1:100	>1:1,600

## Conclusions

In survivors of hantavirus disease who reside in Chile or the United States, we found high titers of plasma NAb against the type of hantavirus that is prevalent in the patient's own region, while substantial titers against the heterologous agent of HCPS were absent. In this small group of participants, NAb titers did not show any readily detectable decline with time elapsed after infection; titers as high as 1:1,600 could be detected 11 years after illness. These results suggest that plasma from patients who survive hantavirus infection is a potential source of NAb and could be used as a therapeutic alternative for patients with acute disease or as a prophylactic intervention for persons who may have been exposed to the virus. The absence of in vitro cross-neutralization makes the alternative of clinically effective cross-protection less likely and discourages the use of convalescent-phase sera to treat patients whose geographic origin is different from that of the plasma donor. Our results suggest that a monovalent vaccine would not elicit protection against different types of hantavirus, even when the viruses are phylogenetically as similar as SNV and Andes virus. The positive results of cross-protection studies in hamster models should be interpreted cautiously, since experimental infection in those studies would tend to favor unusually brisk immune responses that go well beyond eliciting NAb and likely include potent cell-mediated or innate immune responses that cannot be mimicked with passive immunization (12). Similarly, some component of the cross- protective efficacy observed with genetic immunizations with hantavirus envelope genes may ultimately be related to T-cell immunity (13). From this perspective, either multivalent or region-specific vaccines may have to be developed to protect persons at high risk from this new, relatively infrequent, but still highly lethal disease.
